# Anæsthetics

**Published:** 1905-07-22

**Authors:** 


### ANÆSTHETICS.

DUDLEY BUXTON,[1] writing on the clinical aspect of the work of the Chloroform Committee, lays particular stress upon the administration of high-percentage vapour as being the great danger in chloroform anæsthesia. Recent investigations show that a 2-per-cent. vapour is sufficient, as a rule, to induce anæsthesia, which can be maintained with about 1 per cent.; stronger than 3 per cent. should not be used. Deaths occur sometimes at the very commencement of administration. "A few whiffs are taken and the patient dies." These Dr. Buxton regards as parallel with similar deaths in the pre-anæsthetic days, when occasionally the patient just before operation struggled, fainted, and never recovered consciousness. Death in such cases is attributable not to chloroform but to fright. Other deaths occur just at the commencement of the operation; some procedure, such as stretching the sphincter, or even the incision, causes the patient to stop breathing and induces, perhaps, fatal syncope. This is commonly attributed to shock, aggravated by imperfect narcosis. This teaching he considers not only erroneous but dangerous, for the practical outcome of it is that patients are often drenched with a strong vapour in order to rapidly induce deep narcosis before the operation is commenced. Now, experiments show that vagal reflexes of the most dangerous character are not absent and cannot be obliterated in even profound anæsthesia; and, as Embley has shown, reflexes which produce dangerous symptoms in adequate but not profound narcosis do not necessarily destroy the animal, but such reflexes do prove fatal when they take place during profound narcosis. In Dr. Buxton's experience the clinical evidence supporting these physiological investigations is ample. He has often observed in cases where the exigencies of surgery have required very deep narcosis that reflex interference with respiration and circulation have been most serious and called for energetic measures to resuscitate the patient; while under light narcosis such reflex disturbances have been merely transitory and soon recovered from.

He believes that an apparatus which enables the anæsthetist to see the amount of chloroform he is using will go far to prevent such fatalities. From practical experience of its use, he considers that the inhaler devised by Vernon Harcourt works satisfactorily. A weak point in this instrument is the great increase in the percentage of chloroform in the vapour which is produced by any shaking of the bottle, and it is nearly impossible to avoid all movement of the bottle. A modification has been designed whereby the bottle is fixed to a stand, so that no movement is necessary. Some anæsthetists in using the inhaler have intentionally resorted to shaking the bottle when the induction was very prolonged; this is a very dangerous practice, as it is impossible then to know the strength of the vapour, which may have become concentrated much above safety point.

Crouch[2] has abandoned the inhaler after giving it a trial; he found in nine cases that the average induction period was twenty minutes, in two of the cases it was half an hour; this is a serious drawback in busy hospital work. He has found that the attention of the anæsthetist is too much concentrated upon the machine instead of upon the patient. In experienced hands the instrument may be satisfactory, but its *raison d'être* is the exhibition of the drug in such a way that it may give a safe anæsthesia in the hands of the inexperienced practitioner. The majority of anæsthetists at present appear to think that a satisfactory regulating inhaler has not yet been devised.

Hewitt[3] differentiates between "simple" and "complex" chloroform anæsthesia; by the former he means the characteristic state of deep insensibility produced in subjects not undergoing any surgical operation; by "complex," the condition characterised not only by phenomena referable to the chloroform itself, but also by phenomena referable to the surgical procedure which is taking place, he points out that it is necessary to realise the differences between these states in order to understand the nature of the difficulties and accidents that may occur. Many of the phenomena commonly attributed to the anæsthetic are in reality due to the operation. For example, reflex retraction of the tongue, laryngeal spasm, and general respiratory spasm are the result generally of surgical manipulations, and may bring about an asphyxial state quite foreign to chloroform anæsthesia. He lays stress upon the prime importance of maintaining a free air-way for the patient—a matter, in his opinion, deserving more attention than the percentage strength of the vapour administered. He has himself tried and seen in use many appliances for adjusting the strength of the vapour, and on many such occasions he has observed evidences of asphyxia depending either on some condition in the patient himself preventing the free exchange of air, or upon the narrow channels of the inhaler through which the patient has been breathing.

While recognising certain advantages in a percentage inhaler, he thinks that the disadvantages outweigh them. They are cumbrous, complicated, and liable to get out of order. They are, moreover, apt to become fouled, and are incapable of sterilisation. To the man of experience a percentage inhaler is useless, and to the inexperienced man it is complex and requires a scientific attitude of mind not always to be obtained. He does not consider it advisable to induce chloroform anæsthesia with chloroform, but recommends the nitrous oxide-ether sequence until the stage of excitement, the most dangerous time with chloroform, is over.

[1] Brit. Med. Jour., Sept. 24, 1904. [2] Ibid. [3] Lancet, Nov. 26, 1904.

(*To be continued.*)

